# The Power of Humbug

**Published:** 1881-03

**Authors:** 


### THE POWER OF HUMBUG.

An individual who opened a small tavern near the field of Waterloo was frequently questioned as to whether he did or did not possess some relics of the battle, and he invariably and honestly answered in the negative. But he was very poor; and one day while lamenting to a neighbor, not only of his poverty, but the annoyance to which travelers subjected him, his friend cut him short with, "Well, make one help the other. Make some relics."

"But what can I do?" inquired the poor man.

"Tell them that Napoleon or Wellington entered your shop during the battle, and sat down in that chair."

Not long after an English tourist entered the tavern, and, inquiring for relics, was told the chair story. The chair was bought at an incredible price. The next comer was informed that Wellington had taken a drink, and the Wellington tumbler was accordingly sold. The third arrival gazed with breathless wonder at the nail on which Bonaparte had hung his hat; the fourth purchased the door-posts between which he had entered, and the fifth became the happy purchaser of the floor on which he had trodden. At the last advices, the fortunate tavern keeper had not a roof to cover his head, and was sitting on a bag of gold in the corner of a deep pit formed by selling the earth on which the house had stood.

---

THE GOSPEL HISTORY.—A Complete Chronological Narrative woven from the Text of the Four Evangelists. With Notes, original and selected, and Indexes. By James R. Gilmore ("Edmund Kirke"), author of "The Life of Jesus, according to His Original Biographers," and Rev. Lyman Abbott, D.D., author of "Jesus of Nazareth: His Life and Teachings," "New Testament Commentaries," etc. 16mo, 840 pp., cloth, red edges, $1.75. Published by Fords, Howard & Hulbert, New York.

---

A BEAUTIFUL THOUGHT.—To-day, to-morrow, every day, to thousands the end of the world is close at hand. And why should we fear it? We walk here, as it were, in the crypts of life; at times from the great cathedral above us we hear the organ and the chanting choir, we see the light streaming through the open door, when some friend goes out before us; and shall we fear to mount the narrow staircase of the grave that leads us out of this uncertain twilight into eternal life?—*Longfellow.*

